# Location-Privacy Leakage and Integrated Solutions for 5G Cellular Networks and Beyond

**DOI:** 10.3390/s21155176

**Published:** 2021-07-30

**Authors:** Stefano Tomasin, Marco Centenaro, Gonzalo Seco-Granados, Stefan Roth, Aydin Sezgin

**Affiliations:** 1 Department of Information Engineering, University of Padova, 35100 Padova, Italy; marco.centenaro.it@ieee.org; 2 CNIT, the National Inter-University Consortium for Telecommunications, 43124 Parma, Italy; 3 Department Telecommunications and Systems Engineering, Universitat Autònoma de Barcelona, Bellaterra, 08193 Barcelona, Spain; gonzalo.seco@uab.cat; 4 Fakultät für Elektrotechnik und Informationstechnik, Ruhr University Bochum, Digitale Kommunikationssysteme, 44801 Bochm, Germany; stefan.roth-k21@rub.de (S.R.); Aydin.Sezgin@rub.de (A.S.)

**Keywords:** privacy, cellular networks, localization, 5G

## Abstract

The fifth generation (5G) of cellular networks improves the precision of user localization and provides the means to disclose location information to over-the-top (OTT) service providers. The network data analytics function (NWDAF) can further elaborate this information at an aggregated level using artificial intelligence techniques. These powerful features may lead to the improper use of user location information by mobile network operators (MNOs) and OTT service providers. Moreover, vulnerabilities at various layers may also leak user location information to eavesdroppers. Hence, the privacy of users is likely at risk, as location is part of their sensitive data. In this paper, we first go through the evolution of localization in cellular networks and investigate their effects on location privacy. Then, we propose a location-privacy-preserving integrated solution comprising virtual private mobile networks, an independent authentication and billing authority, and functions to protect wireless signals against location information leakage. Moreover, we advocate the continuous and detailed control of localization services by the user.

## 1. Introduction

Our position and movements reveal sensitive information about our lifestyle, preferences, acquaintances, health and work. Over the last twenty years, location-based services (LBSs) for mobile devices were first a subject of study and then became a reality in commercial applications for advertisement campaigns, crowd-sourcing traffic analysis, chatting and augmented reality. The precise localization of devices is also important in the context of the Internet of Things and future industry: indeed, it has been advocated as a key target for the sixth generation (6G) of mobile networks [1]. In 2020, the localization of smartphones was considered for contact tracing in the fight against the coronavirus disease 2019 (COVID-19) outbreak. On that occasion, the privacy of position and movement information was widely debated, and solutions based on ranging (rather than geolocalization) were adopted. Indeed, the unregulated disclosure of position information has both personal and societal impacts that require specific solutions for their mitigation [2].

When considering devices connected to a cellular network, as we will discuss in better detail in the following, the information about user position in a cellular system can be obtained by several entities, all posing privacy issues. First, location is fairly well known to the mobile network operator (MNO), which not only knows in which cell the user is located, but can also obtain the position within the cell with specific techniques. Over the recent years, in several cases, MNOs have disclosed or sold user location information to other parties (see [3] and references therein). Although in some countries stricter regulations may reduce this privacy leakage, suitable technical solutions are useful in unregulated contexts or as an additional security feature. Moreover, the broadcast nature of the wireless medium paves the way for the opportunistic exploitation of radio signals to localize transmitting devices, making the protection of location privacy more problematic. In fact, location information must be protected against its improper use by the MNO, but also by *eavesdroppers* using passive receivers or fake next-generation NodeBs (gNBs) [4]. Moreover, the increasing spread of personal devices that can be used as information sources and the ability to process big data in real time provide the means of inferring user movements from radio signals.

The fifth generation (5G) of cellular networks introduces several innovations in user localization, including smaller cells, millimeter-wave transmissions and powerful in-network artificial intelligence tools for location inference. Moreover, 5G networks can disclose location information to over-the-top (OTT) service providers, further threatening privacy. Location privacy is even more at risk in 6G networks, where sub-millimeter-wave transmissions will provide more accurate localization. Therefore, location privacy is a serious concern in current and future networks [5].

In this perspective paper, we provide an overview of location privacy issues in 5G and beyond cellular networks, and propose an integrated solution to protect location information against its improper use by MNOs, OTT service providers, and eavesdroppers. Our solution includes (a) a virtual private mobility network *VPMN*, which assists end users in concealing their location; and (b) *anonymization* techniques applied on physical-layer signals, which prevent the leakage of location information to eavesdroppers. An independent authentication and billing authority is also introduced, while proper mechanisms give the user more awareness and control over the use of their estimated position. The scope of this perspective paper is twofold: while we highlight several location privacy issues in current cellular networks, we also introduce three approaches to deal with them. Although several technical and possible legal issues must be addressed before a full deployment of our solutions will be possible, we would like to indicate the main directions for the design of a new security architecture. Indeed, we stress that it is possible to protect location privacy only by an integrated approach operating at different levels (single user, group of trusted users and all wireless devices).

## 2. Literature Background

Location privacy has recently attracted the attention of researchers due to the widespread use of mobile devices and the improved localization precision of recent cellular network generations.

Most approaches focus on location information management at network or application layers. In [3], an architecture for the decoupling of the connectivity functionality from the billing and authentication part was considered, and a good privacy solution was proposed, where on-air communications are anonymized and authentication is performed at higher layers through the Internet protocol (IP). Furthermore, location privacy is obtained by exploiting the tracking area lists, enabling users to not disclose the current tracking area. While strengthening the location privacy, this solution still operates at higher layers, leaving open the possibility of localizing and identifying users by physical layer techniques, as detailed in the following. Other approaches resort to k-anonymity [6] or differential privacy [7]. In [8], the direct connection between the location service provider and the user is cut by resorting to a multi-server architecture and a differential privacy approach. In [9], the concept of *mix zones* is introduced, where the connection between the user and the LBS provider is mediated by a middle-ware that provides anonymized location information. A solution based on blockchains is studied in [10], satisfying the principle of k-anonymity privacy protection without the help of trusted third-party anonymizing servers. For a review of anonymization techniques to prevent localization, see [10] and references therein. In [11], a generative adversarial network is used at the MNO to cloak both the location and the trajectory information. However, all these solutions are inadequate for hiding location information from the MNO. In this paper, we instead propose a general architecture protecting location privacy against multiple entities, including the MNO, OTT operators, and external eavesdroppers. In particular, our VPMN solution addresses several privacy issues across various layers.

To ensure location privacy at lower layers, a first solution consists of avoiding the use of identifiers that enable the connection of a user identifier with its location. In [12], attacks operating at layer 2 of the Long-Term Evolution (LTE) standard are discussed, including the tracking of user positions and the mapping of user temporary network identity to the temporary radio identity. Eavesdropping the LTE temporary identifiers also enables the tracking of users as they move from one cell to another [13]. However, anonymization is not enough, as in [14], it was shown that the use of a sequence of different identifiers while moving is still vulnerable to location profiling, as it reveals regularly visited locations. Moreover, by trajectory linking, an attacker can also infer the link among activities of different identifiers, providing a way to identify users.

## 3. Localization Techniques

**Figure 1:**
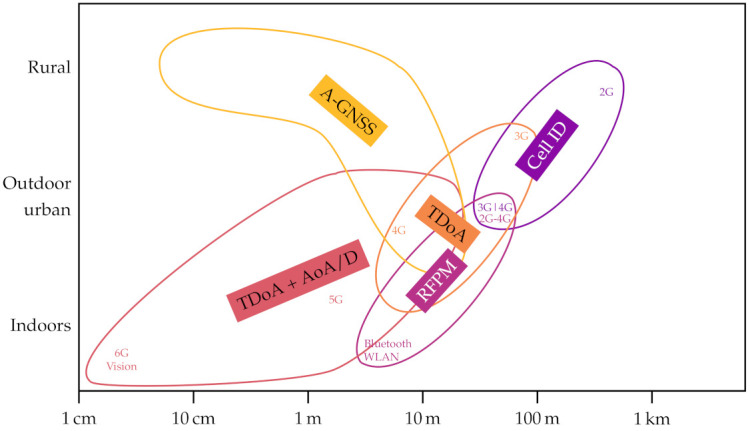
Expected horizontal accuracy of cellular network localization methods for indoor, outdoor urban and rural scenarios.

Localization techniques have become more accurate over time. In the evolution of cellular networks from the first to the fifth generations, a larger bandwidth and the use of multiple antennas has led to better localization. Figure 1 shows the expected horizontal accuracy of various localization methods: the accuracy also depends on the coverage of the localization systems in specific propagation conditions (indoor, outdoor urban and rural).

The first two network generations did not include explicit localization services. Still, typical cell sizes made it possible to narrow the user equipment (UE) position within 1 km of its serving gNB.

The Universal Mobile Telecommunications Service (UMTS) and Long-Term Evolution-Advanced (LTE-A) included the estimation of times of arrival and powers of downlink and uplink signals, which can be used to improve the localization precision below 100 m. Radio frequency pattern matching (RFPM) techniques can further push the limit down to about 50 m. RFPM is based on a) collecting radio channels for given UE positions and b) matching the channel of a UE to the collected database. With time difference of arrival (TDoA) techniques, instead, the times of arrival of downlink or uplink signals provide the differences of distances between the UE and several gNBs, which are then used for trilateration. Thanks to more refined pilot signals and larger bandwidths, TDoA methods reduced location uncertainty from about 100 m in UMTS to a few tens of meters in LTE-A [15].

In 5G networks, the localization accuracy with millimeter-wave transmissions can be on the order of 10 m or less outdoors and 3 m indoors, in part thanks to cooperative gNBs and massive multiple-input multiple-output (MIMO) with angle-of-arrival and angle-of-departure measurements [16]. Going one step further, the 6G vision is to provide decimeter-level accuracy outdoors and one centimeter accuracy indoors by employing, among other solutions, even higher carrier frequencies, intelligent meta-surfaces and artificial intelligence [1].

Note that smartphones can also use other received radio signals for localization purposes. Among these, global navigation satellite systems (GNSSs) can provide centimeter-level accuracy under good satellite visibility conditions when corrections services are used. The transmission of advanced GNSS corrections from the location management function (LMF) to UEs has been included in the 5G standardization [17]. In addition, wireless local area network (WLAN) signals can be used for localization purposes: typically, from the locations of WLAN access points, the receiver location can be established within a few meters or less. A similar approach can be applied to Bluetooth signals, using the known positions of beacons, location units, or other paired devices.

User localization can be performed by (i) the UE itself; (ii) the MNO and OTT providers (legitimately); or (iii) an eavesdropper, overhearing the signals transmitted by the UE, as detailed in the following.

### 3.1. Localization by the User and the Mobile Network Operator

The UE can estimate its own position by processing GNSS signals, possibly with supporting information transmitted by the cellular network, for example, through the cell-specific reference signal (CRS) or the position reference signals (PRS), the latter being available since LTE cellular networks.

As discussed earlier, the MNO can obtain the user position by either the TDoA of uplink signals such as the sounding reference signal (SRS) or RFPM. The obtained position accuracy is significantly improved at higher frequencies by using multiple antennas at the receiver, and possibly simultaneous localization and mapping techniques to locate the scatterers.

GNSS, WLAN, and Bluetooth signals are instead not immediately available to the MNO and still largely remain under the user’s control, as discussed in more detail in Section 9.

### 3.2. Localization by Eavesdroppers

Eavesdroppers may apply various techniques to localize the target UE (see [15] for a survey). The first class of such techniques derives the location information directly from the user, operating at the application layer. Thereby, the eavesdropper gains access to an application running on the UE (either with the consent of an unaware user or as a virus), and the position estimated by the UE is transferred to the eavesdropper by the application via the cellular network and Internet.

The second class of techniques is based on overhearing signals exchanged by the UE with either the cellular network or other wireless devices [18]. Beyond localizing the signal source, the eavesdropper needs also to associate the signal to the target user (i.e., to identify the signal). Signals that can be opportunistically exploited and identified include the cell identification (CID) number (as well as its enhanced version, E-CID), the medium access control (MAC) address exchanged during the handshake with WLANs, and the beacon Bluetooth signals.

Control signals transmitted by the UE can also be exploited for localization purposes. For example, when operating in the frequency-division duplexing mode, the UE periodically sends the downlink channel-state information (CSI) to the serving gNB. This CSI is typically transmitted unencrypted on control-plane physical channels, thus becoming a useful source of information for localization [19]. While with TDoA techniques the receiver needs elaborate signal processing, with CSI eavesdropping the processing is conducted by the UE, and a simple mapping is needed.

As a third class, eavesdroppers can also measure physical information related to the UE position themselves, such as their own channel to the UEs or the received strength of overheard UE signals. These attacks require sophisticated hardware and are much more complicated to implement in practice.

## 4. Location Information Flow

With 5G networks, as discussed above, localization reaches an unprecedented accuracy, particularly when the network operates at millimeter waves. Moreover, the network now includes new protocols for estimating, elaborating, and distributing the location information. Indeed, until LTE, the location information was used mainly for emergency services, retained by the MNOs, and hidden to third parties. Instead, 5G networks may expose it to OTT service providers. Furthermore, the MNO can elaborate the location information with artificial intelligence techniques.

**Figure 2:**
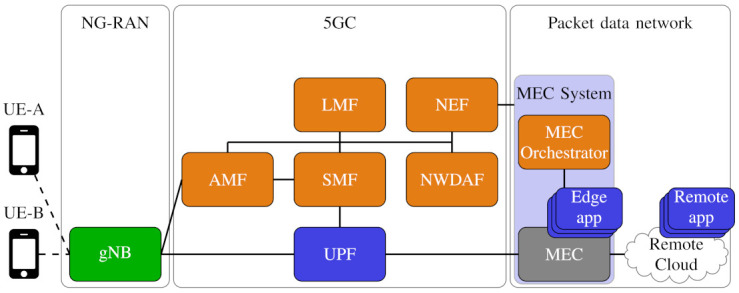
Simplified model of a 5G network with all functions related to localization. Radio access network elements, control-plane network functions, and user-plane network functions are shown in green, orange and blue, respectively. The computing platforms for OTT applications are shown in gray.

We now analyze the flow of location information in order to discover major privacy vulnerabilities. Figure 2 shows a simplified 5G network model, capturing the relevant functions and components related to localization, especially at the 5G core (5GC) level. We will now identify the contribution of each component.

### 5GC Network

The procedure for localization works as follows [17,20]. The *access and mobility management function (AMF)* either receives a request for a location service associated with a particular target UE (e.g., the position estimate for the UE) or may itself initiate a location service (e.g., when the UE is making an emergency call). The AMF then sends a location-service request to the *LMF*, which schedules the resources required for localizing the UE, and may include transferring (receiving) assistance data to (from) the target UE for UE-based (UE-assisted) localization. Lastly, the LMF returns the location information back to the AMF. The *network data analytics function (NWDAF)* operates on data coming from multiple UEs over a long time, thus being able to make powerful data inferences. The NWDAF can exploit (a) the mobility history of the target UE; (b) the mobility history of other nearby UEs; and (c) a potentially huge database of historic positions and movements of UEs in the same area, providing valuable side information on typical movement patterns and derived data (e.g., speeds). NWDAF has been included in the network to infer predictions based on user data: for example, it can predict car traffic evolution by using the location of mobile users. However, NWDAF can also be used to infer the movements of single users. In this context, not only is the target UE position known, but the user interactions with others (up to contact tracing, as in applications to trace COVID-19 contagions) may also be at hand for anyone authorized by the MNO. The *unified data management (UDM)* function is responsible for identifying users and managing network access. Within this, the part responsible for handling the served users is the *session management function (SMF)*.

### Radio Access Network

In the localization process, the *next-generation radio access network (NG-RAN)* may contain multiple *gNBs* and handles various procedures, including the UE localization, the provision of aggregated location-related information, and the delivery of position information between AMF, LMF, and UEs [20]. The AMF may request that the NG-RAN reports the UE location upon the occurrence of specific events, for example, when a UE is in an area of interest [21]. The position measurement procedure is decided by the network operator, and its accuracy varies according to propagation conditions; it also depends on the user, when resorting to UE-assisted positioning.

### Value-Added Services

Lastly, the current or NWDAF-predicted position information may be disclosed to OTT applications through the *network exposure function (NEF)*. If the OTT application is instantiated on a *multi-access edge computing (MEC)* platform, orchestrated by the MNO in proximity of the serving gNB, it can promptly react to the location information received through the NEF. In turn, OTT applications may access the Internet and applications running on a *remote cloud*, with a variety of services, databases and computational facilities to further elaborate the position information. At the 5GC level, the *user plane function (UPF)* provides the data consumed by the OTT application on the MEC.

## 5. Integrated Position Privacy

As outlined, various entities may access the UE location information in 5G networks: the MNO, the OTT service providers and eavesdroppers. All these entities may improperly use the UE location information. Moreover, we have highlighted the main points in the 5G network from which location information can be obtained: the wireless channel (for an eavesdropper); the NG-RAN, NWDAF, and LMF (for the MNO); and the NEF (for OTT providers). Location privacy should be protected at all these points and against all the entities.

**Figure 3:**
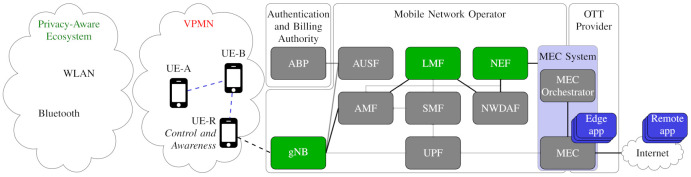
Main components of the location-privacy protection integrated solution, and their interactions. Black lines indicate functional interconnections for the exchange of location information. Dashed blue lines indicate exchanges of messages within the VPMN. Green boxes indicate the control by UE-A of location information in various other network functions.

Next, we propose an integrated solution for the location privacy of cellular networks, whose main components are shown in Figure 3, namely: (a) the *privacy-aware ecosystem*; (b) the *VPMN*, including the independent *authentication and billing authority*; and (c) the *user control and awareness*. Note that, in the spirit of a *perspective paper*, we do not aim to detail the various solutions, but rather to highlight how the approaches complement each other and together address several privacy issues. In Section 9, we also highlight the main technical challenges that need to be addressed by future technical works for a full deployment of the proposed solutions.

**Figure 4:**
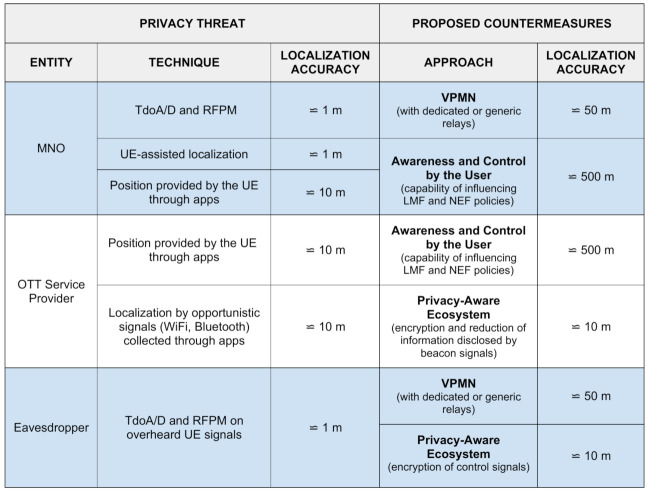
Location-privacy threats and countermeasures.

Figure 4 summarizes the threats and the proposed countermeasures, and indicates the localization accuracy (location privacy) when under attack and when the specific countermeasure is applied.

## 6. The Privacy-Aware Ecosystem

Due to the broadcast nature of the wireless signal, keeping the location of a transmitting device private is a challenging task. Currently, specialized companies [22,23] have collected worldwide databases with billions of positions of WiFi and cell gNBs, as well as fingerprints of their radio signals in various positions. Using the received signal strength and RFPM techniques, they provide accurate positioning within a few meters (in covered areas). This requires the acquisition of the raw radio signal, its elaboration and its comparison with the signals recorded in the database. Therefore, the location is inferred by sensing the electromagnetic environment in the current position. Services also include indoor positioning, in some cases with the deployment of specialized transmitting devices. Simpler approaches may operate at higher layers, by letting an application gain access to the currently sensed WiFi and cellular networks, together with information on the signal strength. A privacy-aware ecosystem refers to a context in which all precautions are taken to protect privacy, preventing such localization techniques. This can be obtained mainly at two levels: technical and regulatory.

At the technical level, in our context, we aim to reduce the transmission or hiding signals that can be opportunistically exploited for localization. Remembering that localization by eavesdroppers also requires the identification of the target UE, all signals transmitted by the UE should be anonymized and location-sensitive signals, such as the CSI-based precoding information, must be encrypted (see also [24], where CSI protection is used for confidentiality purposes). Moreover, Bluetooth beacon signals should be transmitted by the UE only when necessary. Precautions should also be taken when the UE operates as a WLAN hotspot, as it also broadcasts WLAN beacon signals with its ID, offering localization opportunities.

Signals transmitted by other devices can also affect location privacy. Indeed, the WLAN network ID received by the UE can be exploited by an application running on the UE for localization purposes. In this respect, a user that authorizes a specific application to access the WLAN configuration may not be fully aware of the location-privacy implications. Recent versions of Android better protect privacy from such risks, for example, by requiring specific permissions to access the device location in the background, directly accessing WLAN network data and obtaining the precise position by WLAN or Bluetooth [25].

Lastly, technical solutions alone will not ensure location privacy, and should be adequately supported by regulatory actions. The legislator should define both the principles of the accuracy of position information available to the MNO and OTT service providers (regardless of how it is obtained) as well as the technical requirements for the design of future communication systems, not just cellular networks. Indeed, the recent general data protection regulation (GDPR) act in the European Union moves in this direction [26].

### Privacy Improvement

A privacy-aware ecosystem prevents the localization of devices, inferred from the received signal strength or by RFPM techniques. Since these approaches reach an accuracy of a few meters (and below, as millimeter-wave systems are employed), reducing the opportunistic signals (thus preventing such localization) will improve privacy. Considering the presence of legacy devices that will not be updated, location privacy will slowly improve over time with the adoption of the proposed countermeasures. Considering an average lifetime of WiFi routers of about 5 years, the accuracy will be increased to tens of meters in a few years, and will significantly increase within a decade.

## 7. Virtual Private Mobility Network

The main component of the proposed integrated solution is the VPMN. The VPMN is a collection of UEs, wherein each UE can communicate with other VPMN UEs in a device-to-device (D2D) mode, and these VPMN communications are hidden from the gNB. The purpose of this component is to move the privacy protection from the MNO to a group of UEs with mutual trust. Indeed, the VPMN mediates the communication between the cellular network and UEs, whose location is obfuscated to both the MNO and the OTT service providers. Note that here we only propose the new solution of a VPMN, outlining two possible implementations. In this paper we do not have the ambition to address all technical issues related to the deployment of a VPMN, but only to give an overview and indicate possible new research directions. In the next section, we also highlight technical challenges entailed in this solution, which have to be addressed in future technical papers.

We consider two implementations of the VPMN: one with a dedicated *relay* UE in charge of all the communications between the cellular network and the VPMN, and one wherein all VPMN UEs can operate, in turn, as relays.

### 7.1. VPMN with Dedicated Relay

Consider a VPMN, wherein a dedicated UE, denoted *relay* (UE-R), operates as the unique access point between the VPMN and the cellular network.

The uplink transmission from a source UE-A inside the VPMN to the gNB is performed as follows. First, UE-A forwards the packet to another VPMN UE over a D2D communication link; in turn, the receiving UE forwards the packet to another VPMN UE, and the process is repeated until UE-R is reached. Finally, UE-R forwards the packet to the gNB. For the downlink, the VPMN transmits packets to UE-R, which in turn forwards them, possibly through multiple hops, to the destination UE. We now focus on the uplink, while similar considerations hold for the downlink.

The D2D communications should avoid the localization of VPMN UEs and packet tracing by the gNB, as the location of the source UE may be disclosed by these actions. This objective can be achieved by maintaining the D2D communications secret to the gNB. Secrecy can be achieved, for example, by beamforming transmitted signals so that they cannot be decoded by the gNB. Note that VPMN UEs have to know the channel of their link with the gNB, which can be estimated from the downlink *reference signals*. Alternatively, the VPMN establishes an independent (sub)cellular network, thus its communications cannot be overheard by the gNB. In this case, the cellular network may play the role of an eavesdropper, and we must use the previously discussed protection techniques.

### 7.2. VPMN with Generic Relays

Having a dedicated UE operating as a relay reduces the flexibility of the VPMN and drains the energy of the relay. Moreover, if the relay also accesses the network on its own, its location privacy is not protected. Thus, we now consider an alternative configuration, wherein any UE can operate as a relay to the cellular network.

In this case, we must hide the identity of all the VPMN UEs to the cellular network. D2D communications are performed as in the VPMN with a dedicated relay. For relay transmissions, we associate a *temporary ID* to each UE to identify the control plane signals. However, contrary to the temporary ID currently in use in cellular networks, this cannot be associated with the user by the MNO, which would then know the user’s identity. The association is performed by an independent authentication authority connected to the *authentication server function (AUSF)* unit of 5GC that authenticates the VPMN UEs. Possibly, the authority is also in charge of billing users, without disclosing the identity of the UEs to the gNB. Note that authentication and billing functionalities are currently provided by the MNO. The corresponding network function is indicated as the “authentication and billing provider (ABP)” in Figure 3.

In uplink, the relay UE is chosen at random within the VPMN and each packet goes through a multihop secret transmission from the source to the selected relay, before being forwarded to the gNB. In downlink, the VPMN performs an *anycast* transmission to the VPMN UEs, and any UE that decodes the packet then forwards it (possibly by multiple hops) to the intended destination.

Note that in this configuration the location information is partially hidden, as the MNO knows the location of all VPMN UEs, thus the localization uncertainty is within a finite set of known possible positions.

### 7.3. Performance Evaluation

**Figure 5:**
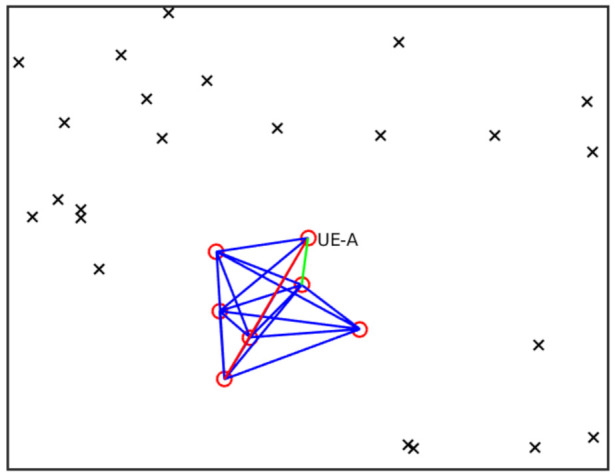
Example VPMN layout. Red circles: UEs of the VPMN. Crosses: other UEs. Green line: minimum distance between UE-A and any other UE in the VPMN. Red line: maximum distance between UE-A and any other UE in the VPMN.

**Figure 6:**
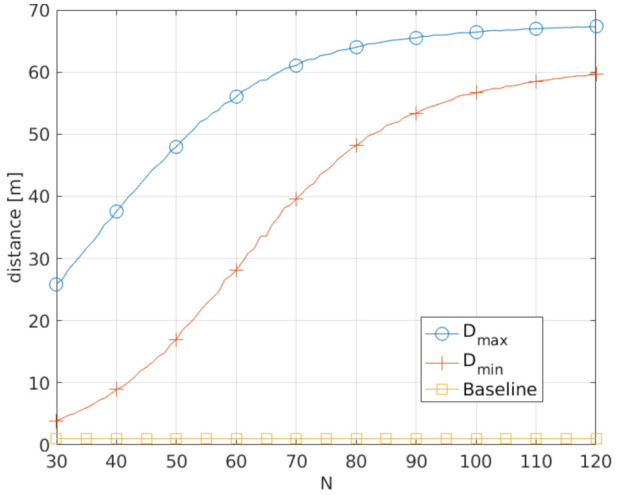
Average maximum and minimum obfuscation distance for UE-A, as a function of the number of UEs *N* in the considered area. *N* UEs are uniformly randomly dropped in an area of 100 m × 100 m. UE-A is at the center of the square area and only UE closer than 15 m communicate directly. The baseline is a distance of 1 m, indicating the position accuracy in the absence of a VPMN.

Consider, for example, that only UEs closer than 15 m may communicate directly. First, we determine the component connected to UE-A, that is, the set of UEs that can be reached by UE-A by multiple hops over other UEs (Figure 5): this will constitute the VPMN for UE-A. As all UEs of the VPMN may operate as uplink relays to the cellular network for UE-A, the distance between UE-A and its farthest VPMN UE represents the maximum uncertainty on the position introduced by the VPMN. Figure 6 shows the average maximum distance *D*_max_ for UE-A as a function of the number of UEs *N*. *D*_max_ grows monotonically with *N* because with more UEs, on average, (a) the connected component gets larger and (b) UEs at a higher distance can be used as relays to the gNB. In the figure, we also report the baseline of 1 m, which indicates the reference accuracy obtained by directly localizing the device when no VPMN is present.

We also require that the position obfuscation is uniform in all directions so that the MNO localizes UE-A only within a disk. To assess the uniformity, we consider three sectors of $120^{\circ }$ and measure the distance of UE-A from its farthest VPMN UE in each sector. Then, the minimum of the three distances provides a measure of the worst-case location obfuscation. Figure 6 shows the average minimum distance *D*_min_ as a function of *N*. For a uniform obfuscation in all directions, we should have *D*_min_ = *D*_max_, which does not occur in practice because of the random position of VPMN UEs, as well as their finite number. Still, in this case, a denser network also yields a larger average minimum distance, thus improving localization privacy.

**Figure 7:**
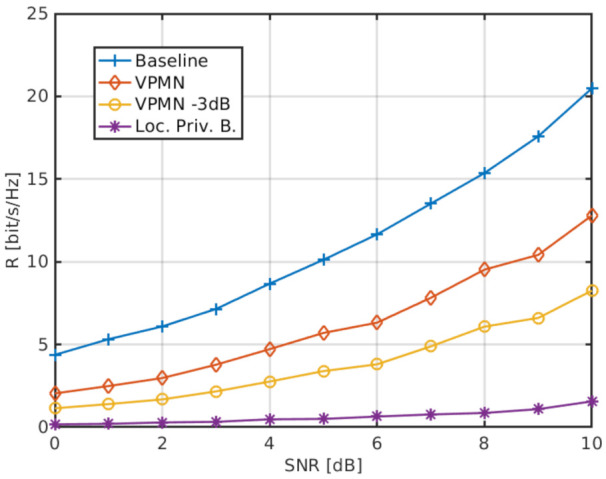
Average achievable rate obtained by the baseline system (no privacy constraints), by the VPMN (with/without 3dB attenuation), and by the location-privacy beamforming of [27], as a function of the average SNR. UEs are equipped with $N_{t}=4$ antennas, UE-A is located at position (0,0), the gNB (with 65 antennas) at (4,0), and UE-B at position (2,2).

We also consider the benefits of the VPMN over the location-privacy beamforming of [27]. Focusing on the scenario of [27], Figure 7 shows the average achievable rates obtained by (a) the baseline system, where UE-A transmits without location-privacy techniques; (b) the VPMN, with transmissions going through UE-B; and (c) the UE-A directly transmitting to the gNB with location-privacy beamforming [27]. While ensuring privacy, location-privacy beamforming yields a significant rate reduction with respect to the baseline system. The VPMN solution instead yields a smaller rate penalty: in this case, the UE-A uses the capacity-achieving beamformers towards UE-B, which hides its location information to the network. To further enhance privacy, we also consider a transmit power reduction of UE-A by 3 dB: this makes localization by gNB more difficult while only slightly reducing the rate, as shown in Figure 7.

VPMN may also improve coverage and connection reliability, similarly to *multipath transmission control protocol (MP-TCP)* [28], wherein transport packets are delivered through multiple paths to increase redundancy. Lastly, note that the VPMN is clearly distinct from a *virtual private network*, as the first hides the device location, while the latter makes data exchange confidential while potentially revealing the device positions.

#### Privacy Improvement

As we can see from these preliminary results, once deployed, the VPMN can reduce the accuracy of localization (at the benefit of privacy) from about 1 m to tens of meters.

## 8. Awareness and Control by the User

In our integrated location-privacy solution, we envision that the UE has a *continuous and detailed control of the user location information*. This includes the possibility for the user to control (a) the access to their location information by each (OTT) service provider; (b) the precision of their location information provided to each network function or OTT application; and (c) the elaboration of their location information by the MNO through the NWDAF. The control must be continuous, with the possibility of easily changing its configuration on a per-application basis at any time.

Currently, a very coarse control is available, with permissions given to applications only at the time of their installation, and the possibility of easily blocking localization services for all the applications at any time. Changing the permissions of each application instead requires more elaborate operations. In Android, the precision of the location information currently has only two levels (coarse and fine [25]) and does not reflect the complexity of scenarios or the power of inference provided by artificial intelligence.

The UE should be able to control which location information is disclosed by gNB, LMF and NEF, as indicated by the green boxes in Figure 3. It is also relevant to increase the user *awareness* regarding the use of location information—not only at the phase of installation of the application, but also during its functioning. Thus, suitable messages should indicate not only when the position information is used by each application, but also the precision of the provided localization.

### Privacy Improvement

The privacy improvement offered by more control given to the user is immediately measurable. Indeed, the accuracy is set directly by the user, according to their preference.

## 9. Technical Challenges

### Privacy-Aware Ecosystem

While it is important to preserve privacy by avoiding the transmission of signals that reveal this sensitive information, broadcast signals, such as beacons, remain important (e.g., for service and device discovery), and thus they cannot be eliminated, but a change of paradigm in their design is needed. While, in the past, these signals have been kept simple for low-cost transceivers, in the future, they should be more robust to privacy attacks. Indeed, this is in line with the current and future computational and communication capabilities of smartphones, and with the user awareness of the relevance of privacy. From a technical standpoint, this means that broadcast signals should be anonymized, and decodable only by authorized users.

### User Awareness

The most relevant challenge is the design of user-friendly interfaces, giving the user powerful control of the location information while making it clear and easy to handle. This would allow, for example, the user to specify if the localization precision should be within a settable distance or other boundaries (e.g., quarter, city, region, or country).

### VPMN Design

The implementation of a VPMN raises several relevant issues that should be addressed by future research activity.

- **Resource allocation**: Resource blocks should be reserved for the VPMN for its local transmission in order to avoid interference with the cellular network. This entails a more elaborate resource allocation by the cellular network and a reduction in the control of resource utilization by the MNO. Moreover, the VPMN should be synchronous with the cellular network. Although some of these issues have been addressed when considering D2D communications in a cellular network, the confidentiality of D2D communications makes their interaction with the cellular network more complex.
- **Connectivity**: Connectivity among the VPMN UEs is limited by the constraint of concealing internal VPMN transmissions to the cellular network. This can be achieved by proper transmit beamforming, but it also imposes limits on the transmit power, and thus on the number of directly connected VPMN UEs. To address this issue, multiple hops over various VPMN UEs may be needed when transferring data from a source UE to a specific *relay* UE in uplink. Similar actions should also be taken in downlink. This increases the communication latency, which is a key performance indicator for 5G cellular networks. Another option is to encrypt local VPMN packets, making them anonymous to the cellular network. In this case, we must prevent the MNO from tracking packets routed within the VPMN, as this may already reveal information on the position of the source UE.
- **Mobility management**: For UEs to remain connected inside the VPMN, handover procedures should be followed in order to remain connected to the MNO. Moreover, when a UE is disconnected from a VPMN due to movements, a new VPMN should be created with other UEs.
- **Power consumption**: As relaying consumes power, it is necessary to ensure a fair use of resources among the VPMN UEs. Lastly, multi-hop transmissions in the VPMN increase latency, which may be problematic for some applications.
- **Scaling issues**: On one hand, having a large VPMN increases the location uncertainty, while on the other hand, several of the previous issues become more problematic. Moreover, in a large VPMN with a single relay, the link between the gNB and the relay UE may become a bottleneck, limiting the data rate; the need to go through a large number of hops may also increase the overall latency. The solution with multiple generic relays is more suitable for a large VPMN, as in practice we can split it into multiple smaller VPMNs. Therefore, a suitable trade-off between complexity and location privacy can be achieved.

## 10. Conclusions

We proposed an integrated solution that addresses location privacy issues in cellular networks, considering different entities interested in knowing locations and various localization techniques. It includes a mixture of technical and legal actions to improve user privacy with localization. From the technical standpoint, a re-design of broadcast signals (e.g., beacons) is needed to hinder their opportunistic use, while the use of a VPMN could be a viable solution for a significant obfuscation of the location to MNOs and OTT service providers. Some of the proposed approaches can be adopted in forthcoming standard releases of 5G, while others require significant changes that may be more easily implementable beyond 5G.

## Data Availability

Not applicable.
